# Spike Proteins of SARS-CoV and SARS-CoV-2 Utilize Different Mechanisms to Bind With Human ACE2

**DOI:** 10.3389/fmolb.2020.591873

**Published:** 2020-12-09

**Authors:** Yixin Xie, Chitra B. Karki, Dan Du, Haotian Li, Jun Wang, Adebiyi Sobitan, Shaolei Teng, Qiyi Tang, Lin Li

**Affiliations:** ^1^Computational Science Program, University of Texas at El Paso, El Paso, TX, United States; ^2^Department of Physics, University of Texas at El Paso, El Paso, TX, United States; ^3^Department of Biology, Howard University, Washington, DC, United States

**Keywords:** ACE2, angiotensin-converting enzyme 2, protein- protein interactions, molecular dynamic, spike protein, SARS, COVID-19, SARS-CoV-2

## Abstract

The ongoing outbreak of COVID-19 has been a serious threat to human health worldwide. The virus SARS-CoV-2 initiates its infection to the human body via the interaction of its spike (S) protein with the human Angiotensin-Converting Enzyme 2 (ACE2) of the host cells. Therefore, understanding the fundamental mechanisms of how SARS-CoV-2 S protein receptor binding domain (RBD) binds to ACE2 is highly demanded for developing treatments for COVID-19. Here we implemented multi-scale computational approaches to study the binding mechanisms of human ACE2 and S proteins of both SARS-CoV and SARS-CoV-2. Electrostatic features, including electrostatic potential, electric field lines, and electrostatic forces of SARS-CoV and SARS-CoV-2 were calculated and compared in detail. The results demonstrate that SARS-CoV and SARS-CoV-2 S proteins are both attractive to ACE2 by electrostatic forces even at different distances. However, the residues contributing to the electrostatic features are quite different due to the mutations between SARS-CoV S protein and SARS-CoV-2 S protein. Such differences are analyzed comprehensively. Compared to SARS-CoV, the SARS-CoV-2 binds with ACE2 using a more robust strategy: The electric field line related residues are distributed quite differently, which results in a more robust binding strategy of SARS-CoV-2. Also, SARS-CoV-2 has a higher electric field line density than that of SARS-CoV, which indicates stronger interaction between SARS-CoV-2 and ACE2, compared to that of SARS-CoV. Key residues involved in salt bridges and hydrogen bonds are identified in this study, which may help the future drug design against COVID-19.

## Introduction

Recently, the Severe Acute Respiratory Syndrome Coronavirus-2 (SARS-CoV-2) is raging throughout the world. This is the seventh member of the Coronaviridae family found which is able to affect human health. Among these seven coronaviruses, four of them (HCoV-229E, HCoV-OC43, HCoV-NL63, HKU1) (Fehr and Perlman, 2015) can only cause mild symptoms, while the other three can cause death-leading diseases. Previously to SARS-CoV-2 that started in 2019, two known respiratory coronaviruses can cause serious respiratory syndromes, that are, the Severe Acute Respiratory Syndrome Coronavirus (SARS-CoV) (Zhao et al., 2013) (broke out in late 2003), and Middle East Respiratory Syndrome Coronavirus (MERS-CoV) (Hagan, 2014) (broke out in 2012). The ability of animal-to-human and human-to-human transmission of the 2003 SARS and the following 2012 MERS, resulted in server pandemics that infected over 8,000 and 2,400 reported infected cases including 774 and 858 death cases, respectively. Compared to SARS-CoV and MERS-CoV, SARS-CoV-2 is causing an even more severe pandemic due to its spreading speed and the population affected. Deep studies comparing the different disease-causing coronaviruses will shed light on the fundamental mechanisms of coronavirus related diseases.

Four main structural proteins are found in coronaviruses, including spike (S), envelope (E), nucleocapsid (N), and membrane (M) proteins. Giving special attention to the S protein, of which the main function is to bind to the receptor Angiotensin-Converting Enzyme 2 (ACE2), and entering the host cell after binding (Hagan and Zandi, 2016). Therefore, the S protein plays a crucial role in the first step of infections for disease-causing coronaviruses. Besides, as reported, the S protein-ACE2 interaction is an easy target for drugs or vaccines. Many efforts have been contributed to investigate S proteins and their receptors, such as ACE2 (Arkhipov et al., 2006; Freddolino et al., 2006; Hagan, 2008; Roos et al., 2010). Even though S proteins of SARS-CoV and SARS-CoV-2 share very similar structures, the binding affinities of S protein and ACE2 of SARS-CoV-2 are much higher than SARS-CoV (Koehl, 2018). This might be the key reason for SARS-CoV-2's faster-spreading speed, comparing to SARS-CoV. In this case, revealing the differences in SARS-CoV and SARS-CoV-2 should provide a deep understanding of how coronaviruses affect human health. Due to the essential role of S proteins, this work reveals some mechanisms of SARS-CoV and SARS-CoV-2's S proteins binding to the ACE2 from biophysics perspectives using multi-scale computational approaches.

Nowadays, computational approaches have been widely implemented to study viruses. Some computational research helped to determine structures of viruses (Li et al., 2012), while other studies focused on revealing mechanisms and functions of viruses (van der Hoek et al., 2004; Glowacka et al., 2011; Xian et al., 2019; Lan et al., 2020; Shang et al., 2020). Among them, very few atomic-level simulations were performed on whole virus structures (Ou et al., 2020), because it is extremely challenging to perform atomic-level simulations on viruses due to their large sizes and complexities (Xian et al., 2019), such problems can be solved in two ways: First, instead of all-atom molecular dynamic simulations, many coarse-grained models (Li et al., 2003; van der Hoek et al., 2004; Glowacka et al., 2011; Lan et al., 2020; Liu et al., 2020; Shang et al., 2020) have been developed and implemented that can selectively capture the main information of residues to perform simulations on viruses. Second, various other computational works focused on particular regions of the viruses, such as the viral capsomer-capsomer interactions (He et al., 2020). In current work, the second method was adapted because this manuscript mainly focuses on the interactions between the coronaviruses' S protein and human ACE2, especially their binding domains.

Numerous experimental studies have been conducted to determine the complex structure of SARS-CoV-2 S protein and ACE2 (Arkhipov et al., 2006; Luan et al., 2020a,b). While many other works focused on the mechanisms of SARS-CoV-2 S protein binding with ACE2 (Hagan, 2008; Zaki et al., 2012; Brielle et al., 2020; Luan et al., 2020b; Zhang et al., 2020). It has been demonstrated that both SARS-CoV and SARS-CoV-2 bind with the ACE2 to enter the host cell (Zaki et al., 2012; Luan et al., 2020b). Then the S protein is primed by serine protease TMPRSS2, which release the S protein subunit S2 to fuse the viral and cellular membrane (Brielle et al., 2020). Then the viral gene hicks into the cell and reproduce more viruses. Therefore, drug design or vaccines against SARS-CoV-2 S protein is a promising direction to provide some protection and treatment against SARS-CoV-2. Besides the experimental studies, various computational studies have been conducted to investigate the interactions between SARS-CoV-2 S protein and ACE2. Some systematic comparison and analysis were performed on SARS-CoV-2 S protein RBD and ACE2 to identify potential intermediate hosts transmitting SARS-CoV-2 to humans (Salas et al., 2019). Some MD simulations revealed that the SARS-CoV-2 S protein-ACE2 binding is more temperature sensitive than SARS-CoV S protein-ACE2 binding (van der Schoot and Bruinsma, 2005). Other computational work studied the binding mechanisms between SARS-CoV-2 S protein and ACE2 from various mammals including human, which explores the host range of SARS-CoV-2 and provide potential novel strategy for drug design (Šiber and Podgornik, 2007; Li et al., 2017) Furthermore, some simulation work found that SARS-CoV-2-ACE2 and SARS-CoV-ACE2 have similar binding energies, but the SARS-CoV-2-ACE2 complex have more contacts and larger interface compared to SARS-CoV-ACE2 complex (Li et al., 2016a). Besides MD simulations, some other approaches, such as AutoDock, are also implemented to study the ACE2 inhibitors which may block the interactions between SARS-CoV-2 and ACE2 (Li et al., 2016b). In this work, we utilize comprehensive approaches to study the SARS-CoV-2-ACE2 interactions from several perspectives such as the electrostatic surfaces, electric field lines and binding forces, etc.

In previous related works, we have successfully analyzed the electrostatic potential distributions and electric field lines around a whole viral capsid (Liu et al., 2020). We have investigated the interactions between viral capsomers for Paramecium Bursaria Chlorella Virus (PBCV-1) (He et al., 2020) and Dengue Virus (Li et al., 2015). The results demonstrated that the multi-scale simulation approaches are appropriate to study protein-protein interactions in viruses which indicates that it is a potentially successful direction to go. Herein, we investigated the interactions between ACE2 and S proteins for both SARS-CoV and SARS-CoV-2.

**Figure 1:**
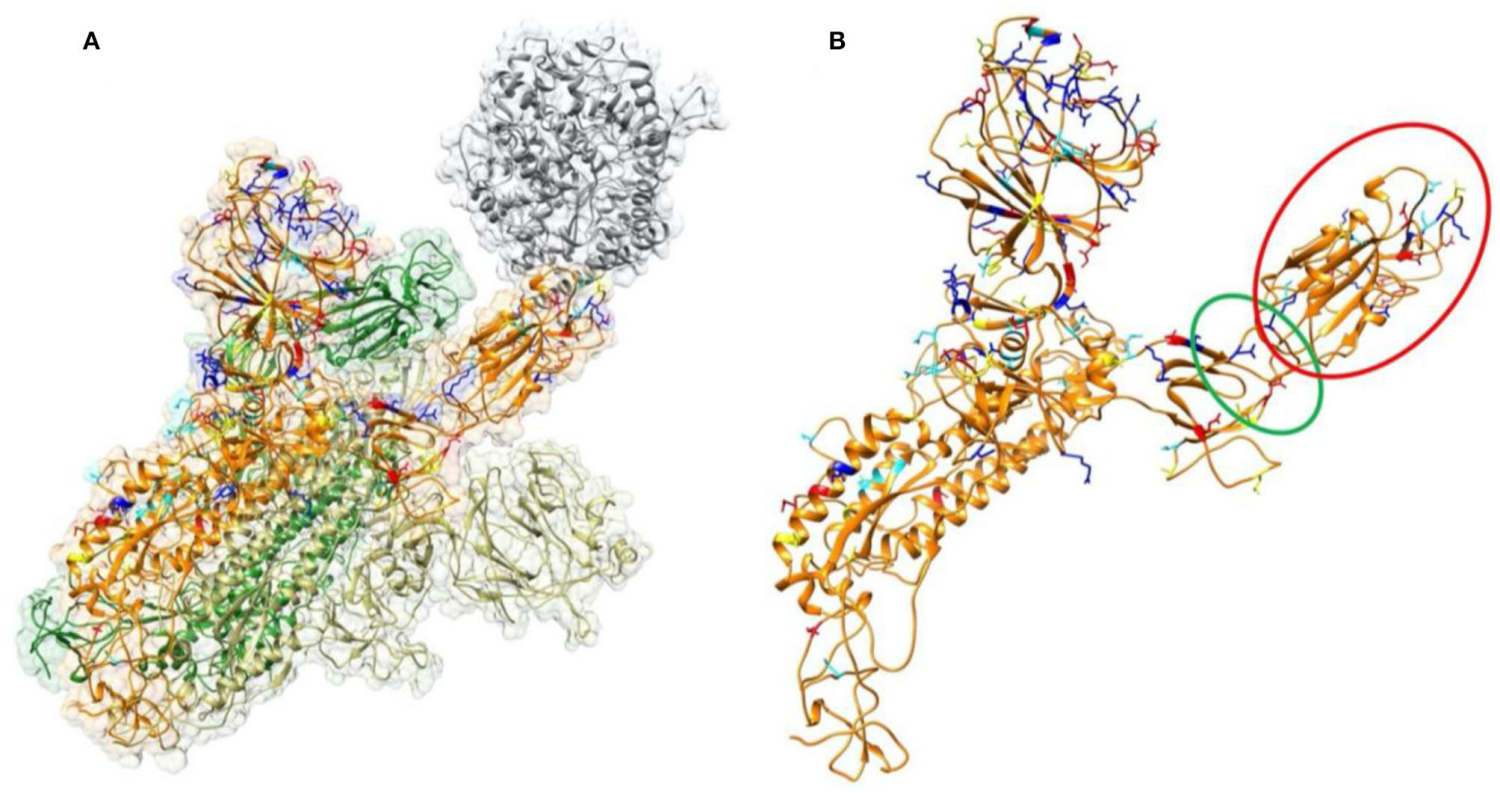
Structure of SARS-CoV-2 S proteins and ACE2 binding domain. **(A)** The structure of S protein trimer binding with ACE2 binding domain. ACE2 is shown in gray color. The three S protein monomers are represented in yellow, orange, and green colors, respectively. The mutations from SARS-CoV to SARS-CoV-2 in this study are labeled in four colors on a single chain of S protein: Red represents residues which are mutated to be more negative; Blue represents residues which are mutated to be more positive; yellow represents residues which are mutated from polar to hydrophobic; cyan represents residues which are mutated from hydrophobic to polar. **(B)** Structure of a single S protein monomer. The RBD shown in red circle is flipping out to reach ACE2. The green circle region highlights the hinge between RBD and the rest of S protein.

The binding interface of electrostatic interaction has been recognized as an important factor for protein-protein recognition and assembly (Phillips et al., 2005; Li, C. et al., 2012; Li et al., 2013, 2017a,b; Li et al., 2020; Wang et al., 2020). Therefore, this study started with the electrostatic features of binding interfaces between ACE2 and S proteins. In our study, several software programs and methods were implemented, including: DelPhi (Nelson et al., 1996; Kumari et al., 2014), DelPhiForce (Biasini et al., 2014; Song et al., 2018), NAMD (Wu et al., 2020), MM/PBSA (Humphrey et al., 1996; Hoffmann et al., 2020), etc. By using DelPhi program, electrostatic surfaces and electric field lines at the binding interfaces were illustrated, which demonstrated ACE2 and S protein RBDs are overall attractive to each other. The differences in electrostatic features between SARS-CoV and SARS-CoV-2 were analyzed. A detailed analysis was performed after MD simulations for both structures separately, which showed that compared to SARS-CoV, the SARS-CoV-2 binds with ACE2 using a more robust strategy. The electric field line related residues are distributed quite differently, which results in a more robust binding strategy of SARS-CoV-2. Also, SARS-CoV-2 has a higher electric field line density than that of SARS-CoV, which indicates stronger interaction between SARS-CoV-2 and ACE2, compared to that of SARS-CoV. This result may explain why SARS-CoV-2 spreads faster and infects a larger population than SARS-CoV. But this study focused on the binding domains of ACE2 and S proteins, which didn't take into account other parts of the S protein. We hypothesized that in the SARS-CoV-2 S protein, the binding domain may be easier to flip out (this region is shown in Figure 1) so that it can interact with the ACE2, as some mutations are found at the linkage between its binding domain and other parts. This mechanism may cause the S protein of SARS-CoV-2 easier to bind to ACE2, which will be studied in our future work. At the end of this manuscript, key residues that are involved in forming salt bridges and hydrogen bonds are identified, which may be considered as targets to help future drug design against COVID-19.

## Methods

### Structure Preparation

The complex structures of SARS-CoV and ACE2 were downloaded from the Protein Data Bank [PDB ID 6ACG (Dolinsky et al., 2004)]. Several SARS-CoV-2 S protein RBD/ACE2 complex structures were determined (Arkhipov et al., 2006; Roos et al., 2010). In this work, we used 6VW1 (Luan et al., 2020b) as our complex structure to study the electrostatic binding interactions between SARS-CoV-2 S protein RBD and ACE2. However, the 6VW1 only contains the binding domains of SARS-CoV-2 S protein and ACE2. To study the electrostatic features for the overall structure of SARS-CoV-2 S protein, SWISS model (Sievers and Higgins, 2014) was used to model the whole structure of the SARS-CoV-2 S protein trimmer binding with ACE2 based on the template of 6ACG. The sequence of SARS-CoV-2 was obtained from Genebank (Maiorov and Crippen, 1994), which was from the early patients in December 2019. The modeled RBD structure has <1 Å RMSD compared to the experimental determined RBD structures, which demonstrates that the modeled RBD structure is very reliable (as shown in Supplementary Figure 1). When studying the electrostatic interactions, we mainly focused on the receptor binding domain (RBD) of S protein and the binding domain of ACE2. To understand the mechanisms of S protein binding to ACE2, S protein RBD was separated from the ACE2 binding domain by a distance from 5 to 40 Å with a step of 1 Å, when analyzing the electrostatic binding forces.

### Electrostatic Calculations Using DelPhi

In order to study the electrostatic features, DelPhi is utilized to calculate the electrostatic potential for the S protein RBD and ACE2 binding domain. In the framework of continuum electrostatics, DelPhi calculates the electrostatic potential ϕ (in systems comprised of biological macromolecules and water in the presence of mobile ions) by solving the Poisson-Boltzmann equation (PBE):

$$\begin{array}{l} \begin{array}{c} \nabla ∙\left[\epsilon \left(r\right)\nabla \phi \left(r\right)\right]=-4\pi \rho \left(r\right)+\epsilon \left(r\right)\kappa^{2}\left(r\right)\sinh\left(\frac{\phi \left(r\right)}{k_{B}T}\right) \end{array} \end{array}$$

where ϕ(*r*) is the electrostatic potential, ϵ (*r*) is the dielectric distribution, ρ (*r*) is the charge density based on the atomic structures, κ is the Debye-Huckel parameter, *k*_*B*_ is the Boltzmann constant, and *T* is the temperature. Due to the irregular shape of macromolecules, DelPhi uses a finite difference (FD) method to solve the PBE.

The electrostatic potential of RBDs of SARS-CoV and SARS-CoV-2 with ACE2 were calculated by Delphi. The calculated electrostatic potential on the surface was visualized with Chimera (**Figure 3**). In order to visualize electric field lines between SARS-CoV and ACE2 and between SARS-CoV-2 and ACE2, Visual Molecular Dynamics (VMD) (Peng et al., 2019) (**Figure 4**) was implemented based on the electrostatic potential map from DelPhi calculations. The color scale range was set from −1.0 to 1.0 kT/Å. In order to clearly show the difference between the surface of SARS-CoV-2 and of SARS, the difference (**Figure 3C**) has been calculated and visualized by subtracting the electrostatic potential values of SARS-CoV-2 by that of SARS-CoV. Since the surface structures are not completely the same, the surface of SARS-CoV-2 was used as the model to visualize the difference of charge distribution (**Figure 3C**).

In the process of DelPhi calculations, the PQR file of each capsomer was generated by PDB2PQR (World Health Organization, 2020). During DelPhi calculations, the resolution was set as 1 grids/Å. The dielectric constants were set as 2.0 for protein and 80.0 for the water environment, respectively. The protein filling percentage of Delphi calculation box (perfil) was set to be 70.0. The probe radius for generating the molecular surface was 1.4 Å. Salt concentration was set as 0.15 M. The boundary condition for the Poisson Boltzmann equation was set as a dipolar boundary condition. The calculated electrostatic potential on the surface was visualized with Chimera (**Figure 3**). VMD was used to illustrate electric field lines between S protein and ACE2 (**Figure 4**). Finally, the color scale range was set to be from −1.0 to 1.0 kT/Å.

### Electrostatic Binding Forces

To compare the strengths and directions of electrostatic forces between RBDs of SARS-CoV-2 and SARS-CoV with ACE2, DelphiForce (Li et al., 2016a, 2017) was implemented to perform the force calculations. As mentioned above, the structures at each distance of S protein and ACE2 protein were used to calculate binding forces. The electrostatic binding forces calculated by DelphiForce were visualized with VMD and represented by arrows. Forces are shown with different S protein RBD-ACE2 distances from 5 to 40 Å with a step of 2 Å. The S protein RBD and ACE2 are separated in the direction of their mass centers connection line. For better visualization of force directions in VMD (Li et al., 2013), arrows were normalized to be of the same size at variable distances, which shows only the direction of each force without considering its strength by sizes. The magnitudes of the electrostatic binding forces were illustrated in **Figure 5** and the trends of forces change of the total binding forces are shown in **Figure 6**.

### Molecular Dynamic (MD) Simulations for SARS-CoV and SARS-CoV-2 RBDs

To simulate the dynamic interactions between S proteins and ACE2 protein, MD simulations were carried out on GPUs using Lonestar5 clusters at the Texas Advanced Computing Center (TACC https://www.tacc.utexas.edu/). A 2,000-step minimization was performed for each simulation, followed by a 100 million steps, during which 20,000 frames were saved from two 100 ns simulations of both SARS-CoV and SARS-CoV-2 separately (1.0 fs per step, 1 frame at each 5000 steps, 100 million steps in total). The RMSDs of the SARS-CoV and SARS-CoV-2 trajectories are about 3.4 and 1.1 Å, respectively (Supplementary Figure 4). During the MD simulations, the temperature was set to be 300 K, and the pressure was set to be standard using the Langevin dynamics. For PME, which is set for full-system periodic electrostatics, with the grid size (86, 88, 132) as (x, y, z) value, respectively. In those two simulations, atoms that are not located in binding domains were constrained within a margin of 10.0 Å of their natural movement maximum length values. In order to get a more accurate result of the simulation, data of the last 50 ns of simulations were selected and used for later data analysis, since the structure of the first 50 ns is not as stable as the last 50 ns of simulations. The simulation processes are visualized in Supplementary Movies 1, 2, generated by VMD.

To analyze the interaction between S proteins and ACE2, the salt bridges that formed within the distance of 4 Å were extracted from the last 10,000 frames of simulations, and for hydrogen bonds the cutoff was 4 Å. The several top-strongest salt bridges in each binding domain were determined by calculating their formation frequency (the occupancy in Supplementary Figure 6) during MD simulation.

## Results

### The Mutations on SARS-CoV-2

To analyze the overall sequence and structural differences between SARS-CoV and SARS-CoV-2, the sequences of SARS-CoV and SARS-CoV-2 studied in this work are aligned using clustal omega (Yan et al., 2020). The result is shown in the Supplementary Figure 2. The positions of those mutations are mapped to SARS-CoV-2 structure as labeled in in four colors (Figure 1) on a single chain of S protein: Red represents residues which are mutated to be more negative; Blue represents residues which are mutated to be more positive; yellow represents residues which are mutated from polar to hydrophobic; cyan represents residues which are mutated from hydrophobic to polar. It is found that most of the mutations distribute on the surface of the S protein. We observed that the mutations in the RBD region (red circle of the Figure 1B) locate close to the interface by facing to the ACE2. This observation indicates that the mechanisms of S protein binding to ACE2 between SARS-CoV and SARS-CoV-2 may be quite different. Therefore, we performed comprehensive analysis of the binding interfaces to investigate their different binding mechanisms. Furthermore, it is obvious that some mutations are located on the hinge, which links the RBD and other regions of the S protein, as shown in the green circle of Figure 1B. It suggests that the flexibility of the RBD may also be different between those two viruses, which might open an avenue for our future research on coronaviruses.

### Electrostatic Surfaces and Field Lines

**Figure 2:**
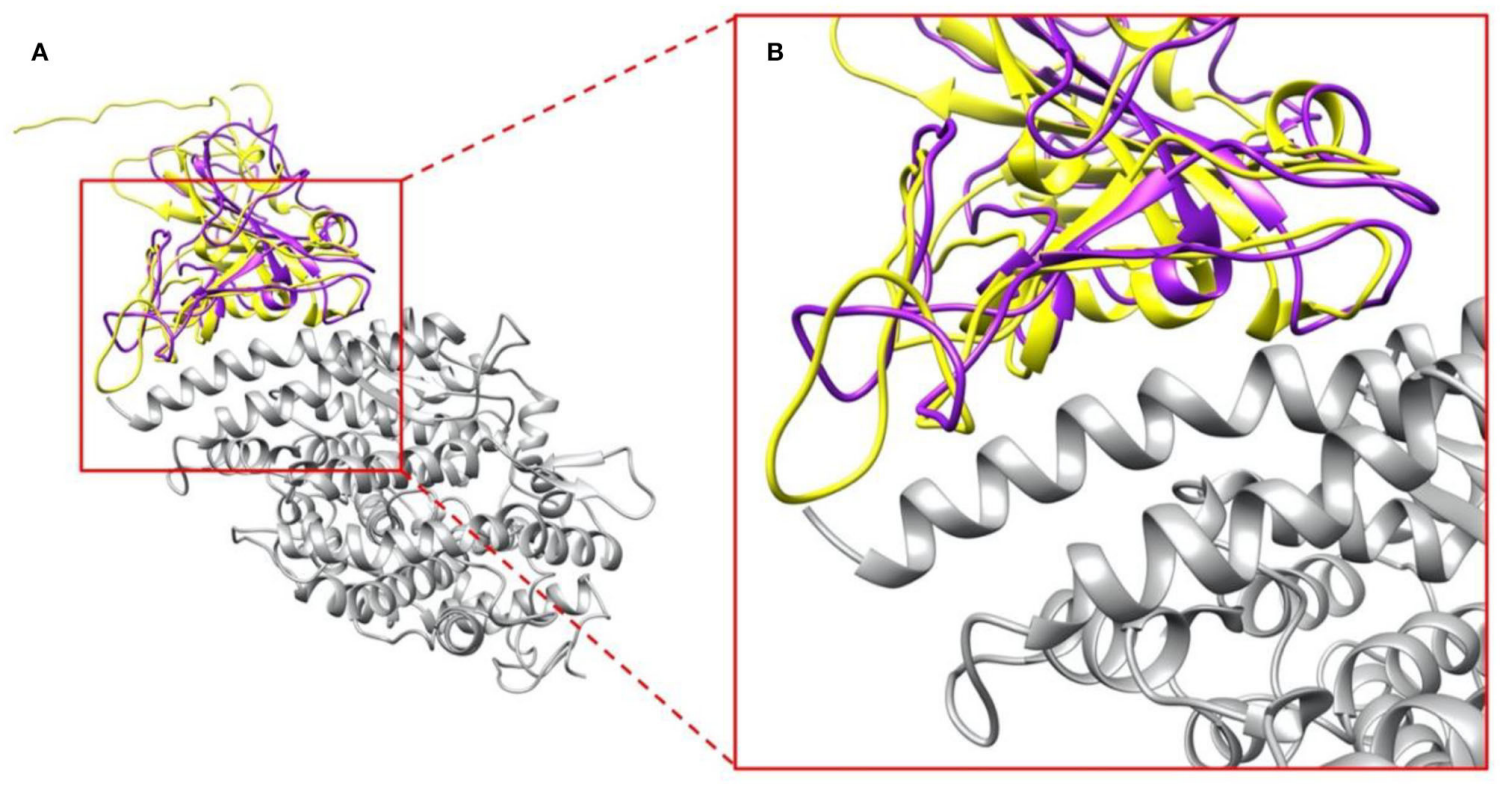
Complex structures of S protein RBDs and ACE2 protein. **(A)** SARS-CoV S protein RBD (purple) and SARS-CoV-2 S protein RBD (yellow) bind with human ACE2 binding domain (gray); **(B)** A closeup view of **(A)**, the interface area of SARS-CoV S protein RBD (purple) and SARS-CoV-2 S protein RBD (yellow) with human ACE2 binding domain (gray).

We compared the structure of the S protein RBDs of SARS-CoV with the same part of SARS-CoV-2. As shown in Figure 2A, SARS-CoV S protein RBD (purple) and SARS-CoV-2 S protein RBD (yellow) are aligned based on their structures, while human ACE2 binding domain (gray) are bound with S protein RBDs. The overall RBDs structures of SARS-CoV and SARS-CoV-2 S proteins are very similar, with the RMSD (Wang, Q. et al., 2020) of 0.965 Å, but some differences can still be noticed in several loops of the RBDs (Figure 2B), which is due to two factors: (1) The high flexibility of the loops; (2) The amino acid differences between SARS-CoV and SARS-CoV-2. The variation of binding mechanisms between the two viruses could be caused by the differential residues rather than the whole structures.

### Electrostatic Surfaces

**Figure 3:**
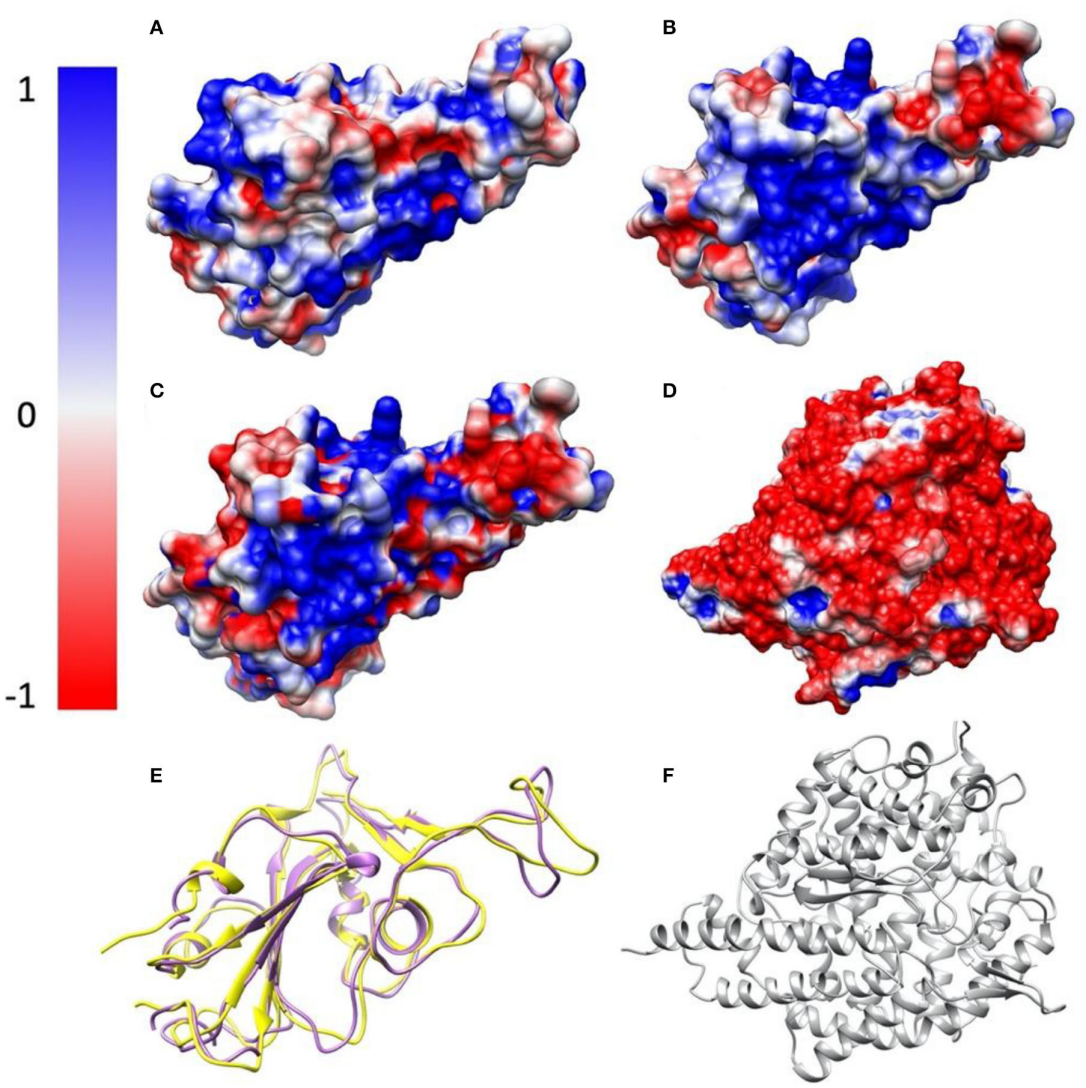
Electrostatic surfaces of SARS-CoV S protein RBD, SARS-CoV-2 S protein RBD and ACE2 RBD. **(A)** Electrostatic surface of SARS-CoV S protein RBD; **(B)** Electrostatic surface of SARS-CoV-2 S protein RBD; **(C)** Electrostatic difference between SARS- CoV and SARS-CoV-2 S protein RBD, by subtracting electrostatic values of SARS-CoV-2 by SARS-CoV, and mapped the values on the surface of SARS-CoV-2; **(D)** Electrostatic surface of human ACE2 RBD; **(E)** Structure comparison of SARS-CoV S protein RBD and SARS-CoV-2 S protein RBD, colored with purple and yellow, respectively; **(F)** The structure of human ACE2 binding domain, colored with gray.

To study the electrostatic features, DelPhi is utilized to calculate the electrostatic surfaces of the S protein RBDs and ACE2. The charge distribution on SARS-CoV S protein RBD is showed in Figure 3A and Supplementary Movie 3 rendered by Chimera, with a color scale from −1.0 to 1.0 kT/Å. The charge distribution on SARS-CoV-2 S protein RBD is shown in Figure 3B and Supplementary Movie 4 rendered by Chimera, with a color scale from −1.0 to 1.0 kT/Å. Negatively and positively charged areas are colored in red and blue, respectively. The electrostatic surfaces shown that the binding interface of ACE2 is dominantly negative, while the binding interfaces of S protein RBDs are all dominantly positive (Figure 3D and Supplementary Movie 5).

The difference of the electrostatic potential (which are generated from DelPhi) between SARS-CoV and SARS-CoV-2 S protein RBDs was calculated and mapped on the surface of SARS-CoV-2, as shown in Figure 3C. From the presentation in Figure 3C, an area of positive charge is observed, which also shows that SARS-CoV-2 S protein RBD is more attractive than the SARS-CoV to ACE2, since ACE2 has an overall negative charged surface, as shown in Figure 3D. Therefore, we expect the SARS-CoV-2 S protein RBD may form more non-covalent bonds with ACE2, such as hydrogen bonds and salt bridges. In the later sections we demonstrate that besides salt bridge residue pairs, the SARS-CoV-2 utilizes a cluster of residues to interact with ACE2, which is more robust than individual salt bridges.

### Electric Field Lines

**Figure 4:**
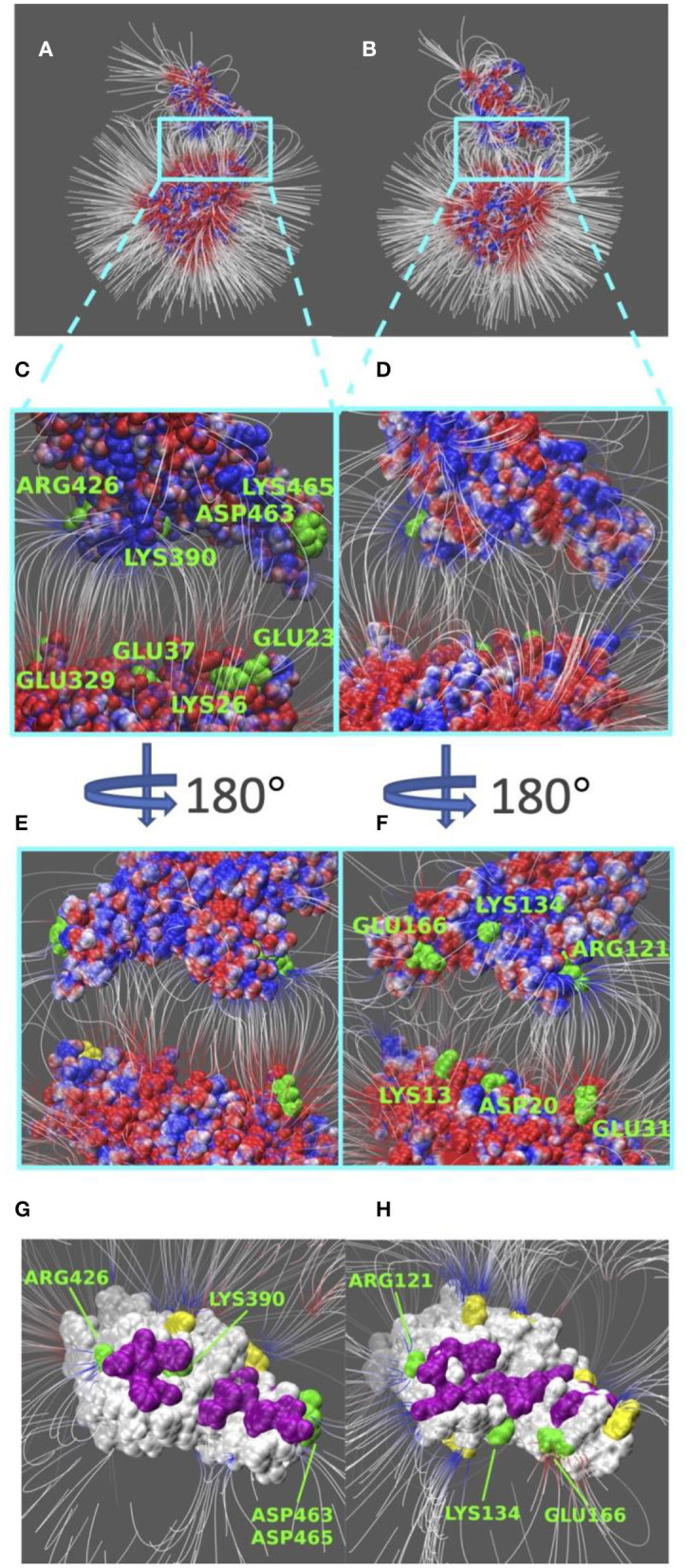
Electric filed lines at the interfaces of S protein RBDs and ACE2. **(A)** An overview of electric filed lines between SARS-CoV S protein RBD and ACE2 RBD; **(B)** An overview of electric field lines between SARS-CoV-2 S protein RBD and ACE2 RBD; **(C)** A closeup view of **(A)**, with marked key residues that form salt bridges (ARG426-GLU329, LYS390-GLU37, ASP463-LYS26, LYS465-GLU23); **(D)** A closeup view of **(B)**; **(E)** The back view of **(C)**; **(F)** The back view of **(D)** with marked key residues that form salt bridges (GLU166-LYS13, LYS134-ASP20, ARG121-GLU31). The electrostatic surfaces and field lines are rendered by Visual Molecular Dynamics (VMD) (Li et al., 2013) with a color scale from −1.0 to 1.0 kT/Å. To present field lines in the clearest way, we adjusted gradient values to 2.39 kT/(eÅ), in **(A,B, E,F)**, and 2.08 in **(C,D,G,H)**. Negatively and positively charged areas are colored in red and blue, respectively; **(G)** The bottom view of SARS-CoV S protein RBD, salt bridge involved residues are marked green, hydrogen bond involved residues are marked purple, and yellow regions are special residues that have high density of field lines but they are not involved in salt bridges nor hydrogen bonds; **(H)** The bottom view of SARS-CoV-2 S protein RBD, salt bridge involved residues are marked green, hydrogen bond involved residues are marked purple, and yellow regions are special residues that have high density of field lines but they are not involved in salt bridges nor hydrogen bonds.

Electric field lines that surround the binding interfaces are calculated using Delphi. To better visualize the field lines between interfaces and show its interaction area with a clear representation, S protein RBDs were separated from ACE2 by 20 Å (Figure 4). In Figure 4, densities of field lines represent the strengths of interactions. Higher density indicates stronger interaction in the region.

**Figure 5:**
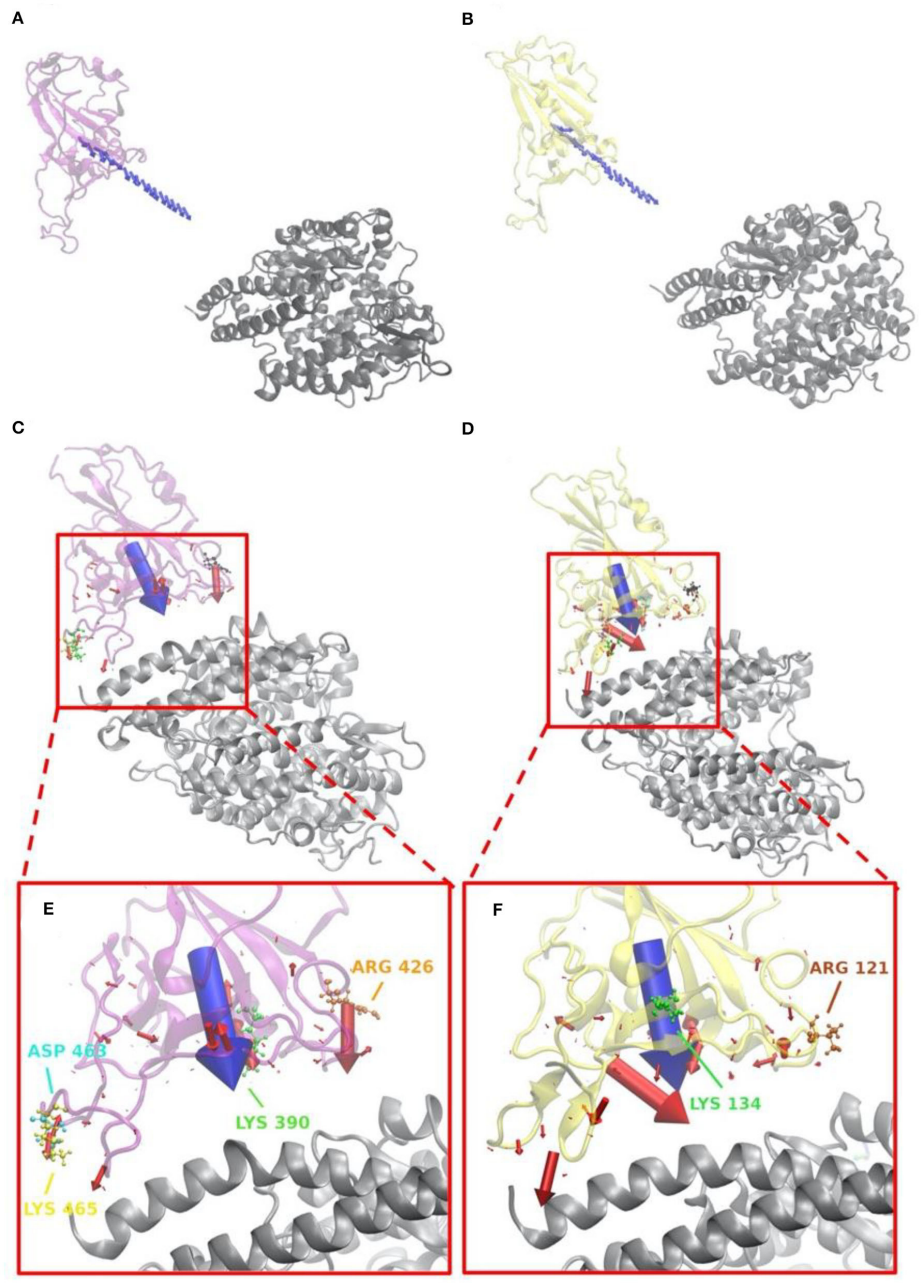
Electrostatic forces of SARS-CoV S protein RBD and SARS-CoV-2 S protein RBD at variable distances with human ACE2 binding domain. **(A)** Electrostatic forces of SARS-CoV S protein RBD with human ACE2 RBD at a different distance, from 5 to 40 with a step of 2 Å, where blue arrows show the net force directions. **(B)** Electrostatic forces of SARS-CoV-2 S protein RBD with human ACE2 RBD at different distance, from 5 to 40 with a step of 2 Å, where blue arrows show the net force directions. **(C)** Electrostatic forces of SARS-CoV S protein RBD with human ACE2 RBD at a distance 5 Å, where the blue arrow shows the total net force between S protein and ACE2, and red arrows represent individual forces between single residues of S proteins in interface area and ACE2. **(D)** Electrostatic forces of SARS-CoV-2 S protein RBD with human ACE2 RBD at a distance of 5 Å, where the blue arrow shows the total net force between S protein and ACE2, and red arrows represent individual forces between single residues of S proteins in interface area and ACE2. **(E)** A closeup view of **(C)** in the interface. **(F)** A closeup view of **(D)** in the interface.

Shown in Figures 4A,B, we see the similarity in field lines of SARS-CoV and SARS-CoV-2 complex structures. In those two panels, the field lines that connect S proteins and ACE2 are clearly shown with high densities all around the surfaces. This fact shows that both SARS-CoV and SARS-CoV-2 S protein RBDs have strong attractive binding forces to ACE2 protein, and the further discussions on binding forces are in the later section of electrostatic forces (Figure 5).

However, there are still several remarkable differences if we take a closer look at the interface areas, as shown in Figures 4C–H. The first difference is the distribution dissimilarity of electric field line related residues. The residues forming salt bridges are distributed differently in SARS-CoV (Figures 4C,E) compared to SARS-CoV-2 (Figures 4D,F). The salt bridge residues of SARS-CoV are clearly shown in the front view of the complex (Figure 4C). In contrast, the salt bridge residues of SARS-CoV-2 are mainly observed in the back view of the complex (Figure 4F). This indicates that the salt bridge residues are distributed on the opposite sides of the S protein RBDs for SARS-CoV and SARS-CoV-2. Besides the front and back views, we also rendered the bottom views of SARS-CoV (Figure 4G) and SARS-CoV (Figure 4H) with colorful patches, where green patches represent salt bridge residues, purple patches represent hydrogen bonds, and yellow patches represent special regions that form high-density field lines but do not belong to salt bridges nor hydrogen bonds. By comparing those patches, SARS-CoV-2 (Figure 4H) has a bigger and more joint hydrogen bond distribution (purple patches) than SARS-CoV (Figure 4G); salt bridges(green patches) in SARS-CoV-2 (Figure 4H) are more concentrated in the distribution, while salt bridges(green patches) in SARS-CoV-2 (Figure 4G) are distributed more separately; and SARS-CoV-2 (Figure 4H) has 5 major special regions (yellow patches), while SARS-CoV (Figure 4G) has only 2 major special regions (yellow patches). The second difference is about density. Figures 4G,H have the same representation setting of field lines with the gradient values of 2.39 kT/(eÅ), it is obvious that SARS-CoV-2 (Figure 4H) has several higher-density field line regions than SARS-CoV (Figure 4G). Since the higher density indicates the stronger interactions, SARS-CoV-2 definitely has stronger attractive interaction than SARS-CoV.

### Electrostatic Forces

Electrostatic forces of SARS-CoV and SARS-CoV-2 S protein RBDs at different distances with human ACE2 binding domain are calculated by DelPhiForce to model the binding forces when S proteins bind to ACE2 (Figure 5). Arrows in Figures 5A,B are shown to visualize the net forces between proteins by shifting the S proteins away from ACE2 by a distance ranging from 5 to 40 with a step of 2 Å. The directions of arrows represent the force directions at different distances. To better visualize the directions of the net forces, the magnitudes of net forces are normalized to be of the same size at different distances, which means that the size of arrows do not represent the force strength. Comparing Figures 5A,B, as we expected, the overall binding forces are all shown to be attractive for both viruses. As for the force directions, only slight differences were found at different distances. From Figures 5C,D that represent the force on every residue in the RBDs at a distance of 5 Å, with the arrow sizes representing the force magnitudes, a conclusion can be drawn that SARS-CoV-2 has quite a different force distribution on individual residues with SARS-CoV. A closeup view of the difference is noticed by comparing Figures 5E,F. The salt bridge involved residues are labeled in the Figures 5E,F, which confirms that the salt bridge residues do provide significant attractive forces in the interaction process.

**Figure 6:**
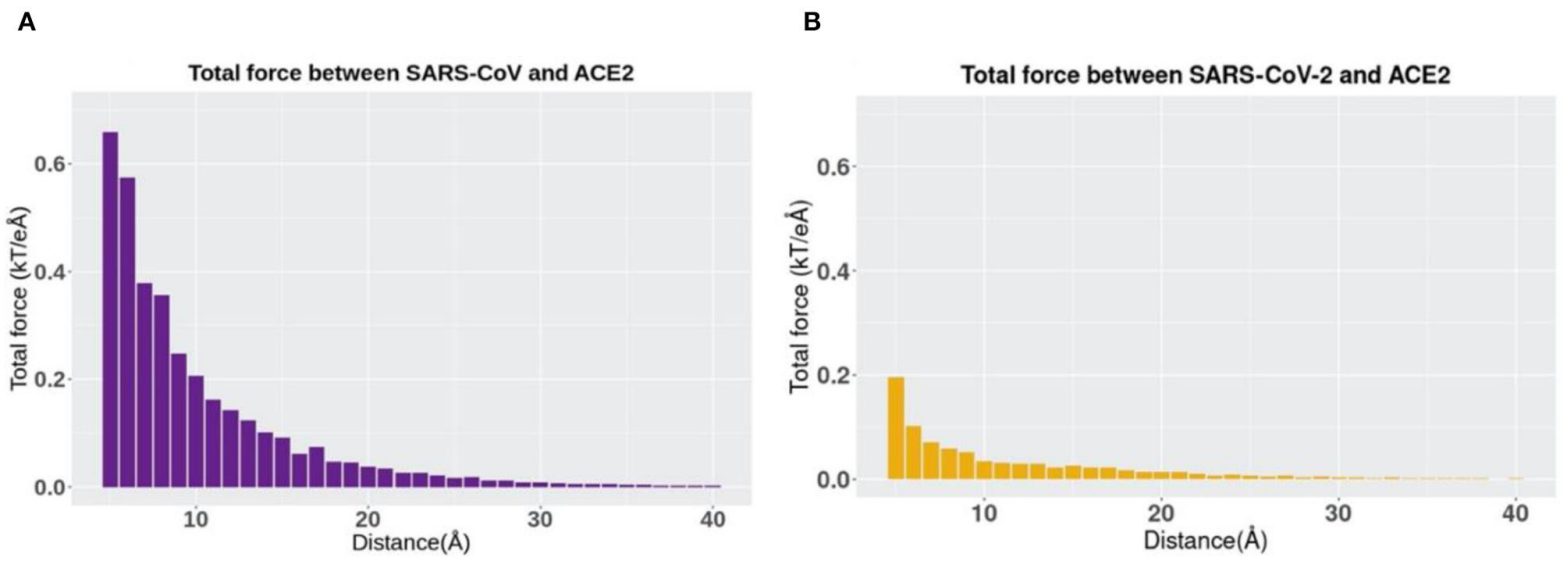
The trends of total electrostatic forces between S protein RBDs and human ACE2 RBD at different distances from 5 to 40 Å. **(A)** Total electrostatic force between SARS-CoV S protein RBD and human ACE2 binding domain. **(B)** Total electrostatic force between SARS-CoV-2 S protein RBD with human ACE2 RBD.

The directions of the net forces are shown in Figures 5A,B, while the magnitudes of the net forces are shown in Figure 6. The magnitudes of the net forces on the directions of mass center lines, x, y, z directions are shown in Supplementary Figure 3. For both SARS-CoV-2 and SARS-CoV, the net forces are enhanced when the distance is decreased from 40 to 5 Å, which is expected to see because the main force is a type of electrostatic force. Based on the Coulomb's law, when the charges on the RBD interfaces get closer to the charged residues on the interface of ACE2, the force increases significantly. Besides, by comparing Figures 6A,B, the net force of SARS-CoV S protein RBD is actually stronger than that of SARS-CoV-2, which might be due to the charge distribution differences between those two binding domains. Even though the attractive force is weaker, the SARS-CoV-2 may still be easier to bind with ACE2. Because there are sequence differences at the hinge which connects the RBD and other parts of the S protein (Figure 1). Such sequence differences may make the RBD more flexible and easier to open and bind with ACE2. We will study the flexibilities of the RBDs from SARS-CoV and SARS-CoV-2 in future work.

Salt-bridge-involved residues on SARS-CoV RBD are marked with different colors in Figure 5C and labeled with their residue types and sequence numbers in Figure 5E. As shown in Figure 5E, for SARS-CoV RBD, four salt bridge residues, ARG426 (orange), ASP468 (cyan), LYS390 (green), and LYS465 (yellow), are labeled. Among them, ARG426 provides a strong attractive force to ACE2 while LYS465 results in a repulsive force to ACE2, due to the fact that the LYS465 faces a positively charged region at the ACE2 interface. However, a negatively charged residue, ASP463 (yellow), is located in a neighborhood which results in attractive force that overcome the repulsive force from LYS465. Also, as shown in Supplementary Figure 6C, the h-bond formed by LYS465 has a 52.32% appearance occupancy and ASP463 has 48.50%. These results indicate that even though LYS465 has repulsive force to ACE2, still the nearby region has attractive force to ACE2. Note that this calculation is based on the structure of S protein separated from ACE2 by 5 Å. When S protein binds to ACE2, the sidechain of LYS465 on S protein changes the configuration to form a salt bridge with GLU23 on ACE2, which is demonstrated in the later section of salt bridges.

Salt bridge residues of SARS-CoV-2 RBD are marked with different colors in Figure 5D and labeled with their residue types and sequence numbers in Figure 5F. For SARS-CoV-2 RBD, two strong salt bridge residues ARG121(brown) and LYS134(green) are observed. As the red arrows shown in Figure 5F all have the direction pointing to ACE2, we can conclude that those two residues are all attractive to ACE2, among which LYS134 has a stronger attractive force strength based on the comparison of arrow sizes.

In terms of the total electrostatic forces between S protein RBDs and human ACE2 RBD, it should be noticed that SARS-CoV-2 has relatively lower values than SARS-CoV, especially when the distance has a smaller value. Note here that in this comparison, we only take the force strength into consideration rather than the directions of forces, and directions can also play an essential role in the comparison.

### Salt Bridges

Salt bridges at the interfaces of S protein RBDs and ACE2 are analyzed based on the MD simulation results and shown in Supplementary Figure 6. Four pairs of salt bridges have been observed between SARS-CoV RBD and ACE2 RBD, comparing to two pairs between SARS-CoV-2 RBD and ACE2 RBD. Among the four pairs of SARS-CoV salt bridges, as shown in Supplementary Figures 6A–D, three of them (ASP463–LYS26, GLU23–LYS465, GLU329–ARG426) are strong salt bridge pairs during 50–100 ns time duration, as the distance is all below 4 Å, which is the selected cut-off value for salt bridge calculations; while the fourth salt bridge (GLU37–LYS390) performs interestingly: at the beginning, GLU37 and LYS390 keep a distance of about 6 Å, from 73 ns, it suddenly becomes the strongest pair with the shortest distance (about 2.75 Å) among those four observations. This change is due to the side chain flexibilities.

Speaking of the two observations of SARS-CoV-2 salt bridges, as shown in Supplementary Figures 6E,F, they are all strong pairs (ASP20–LYS134 and GLU311–ARG121) during the whole 50 ns. While there is a special pair (GLU166–LYS13) which has been observed that is included in Supplementary Figure 5. This special pair has a strong salt bridge feature during the first 30 ns of the whole 100 ns simulation, while those two residues apart from each other to a distance over 7.5 Å after 30 ns. Since we only took the last 50 ns for our data analysis, this special pair is not considered as a salt bridge pair in this work. However, we can still draw a conclusion that some residues involved in the binding process between SARS-CoV-2 and ACE2 are flexible.

### Hydrogen Bonds

**Figure 7:**
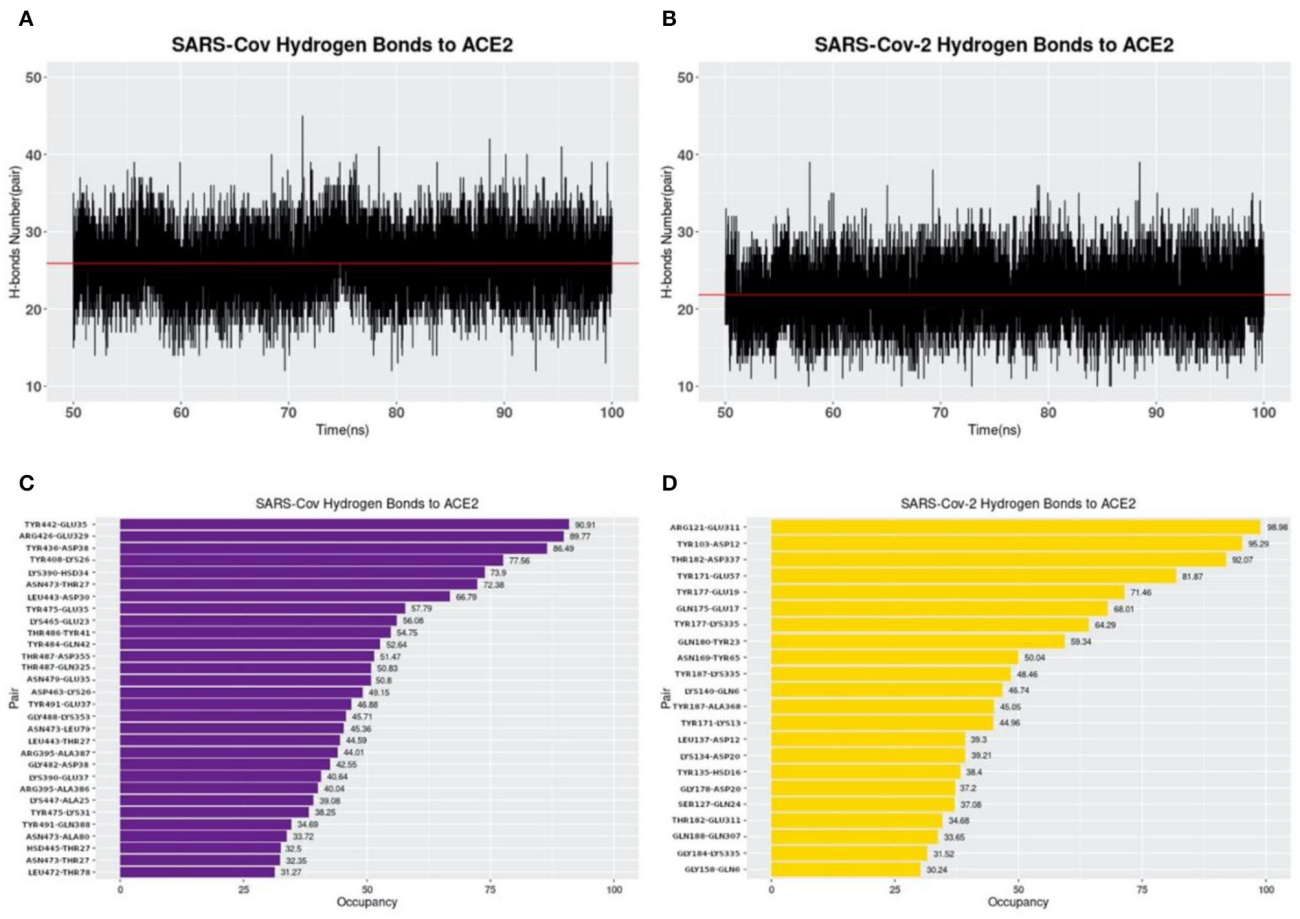
Hydrogen bonds at interfaces of S protein RBDs and ACE2 RBD with their occupancies. **(A)** Number of hydrogen bonds between SARS-CoV S protein RBD and ACE2 binding domain during the MD simulation. The average number of hydrogen bonds is shown as a red line, with the value of 25.90 pairs. **(B)** Number of hydrogen bonds between SARS-CoV-2 S protein RBD and ACE2 binding domain in the MD simulation. The average number of hydrogen bonds is 21.85, shown as the red line; **(C)** Occupancies of 30 pairs of hydrogen bonds (with a cutoff value of 30%) forming at the interface of SARS-CoV S protein RBD and ACE2 protein binding domain. **(D)** Occupancies of 22 pairs of hydrogen bonds forming at the interface of SARS-CoV-2 S protein RBD and ACE2 protein binding domain. For each hydrogen bond pair, the residue on the left is from S protein RBD while that on the right is from ACE2.

Hydrogen bonds at the interfaces of S protein RBDs and ACE2 are also calculated based on the MD simulations as shown in Figure 7. By comparing Figures 7A,B, the average numbers of hydrogen bonds at the same time between SARS-CoV and SARS-CoV-2 S protein RBDs and ACE2 are very similar, with the mean values of 25.90 and 21.85, respectively (marked as the red lines). While by comparing Figures 7C,D, the details of hydrogen bonds with the occupancies above 30% are quite different. The first difference to notice is the highest occupancy of each complex structure, where we find that SARS-CoV-2 has the highest occupancy of 98.98%, compared to 90.91% for SARS-CoV. Besides, if you pick the 90% as a cutoff value, SARS-CoV-2 has 3 pairs, compared to only 1 pair in SARS-CoV analysis, which means SARS-CoV-2 has more extremely high occupancy hydrogen bonds than SARS-CoV. This fact is also an evidence to show the more robust binding strategy of SARS-CoV-2. And it might be another reason why the COVID-19 is spreading easier and faster than SARS in 2003.

### Key Residues Involved in Salt Bridges and Hydrogen Bonds

**Figure 8:**
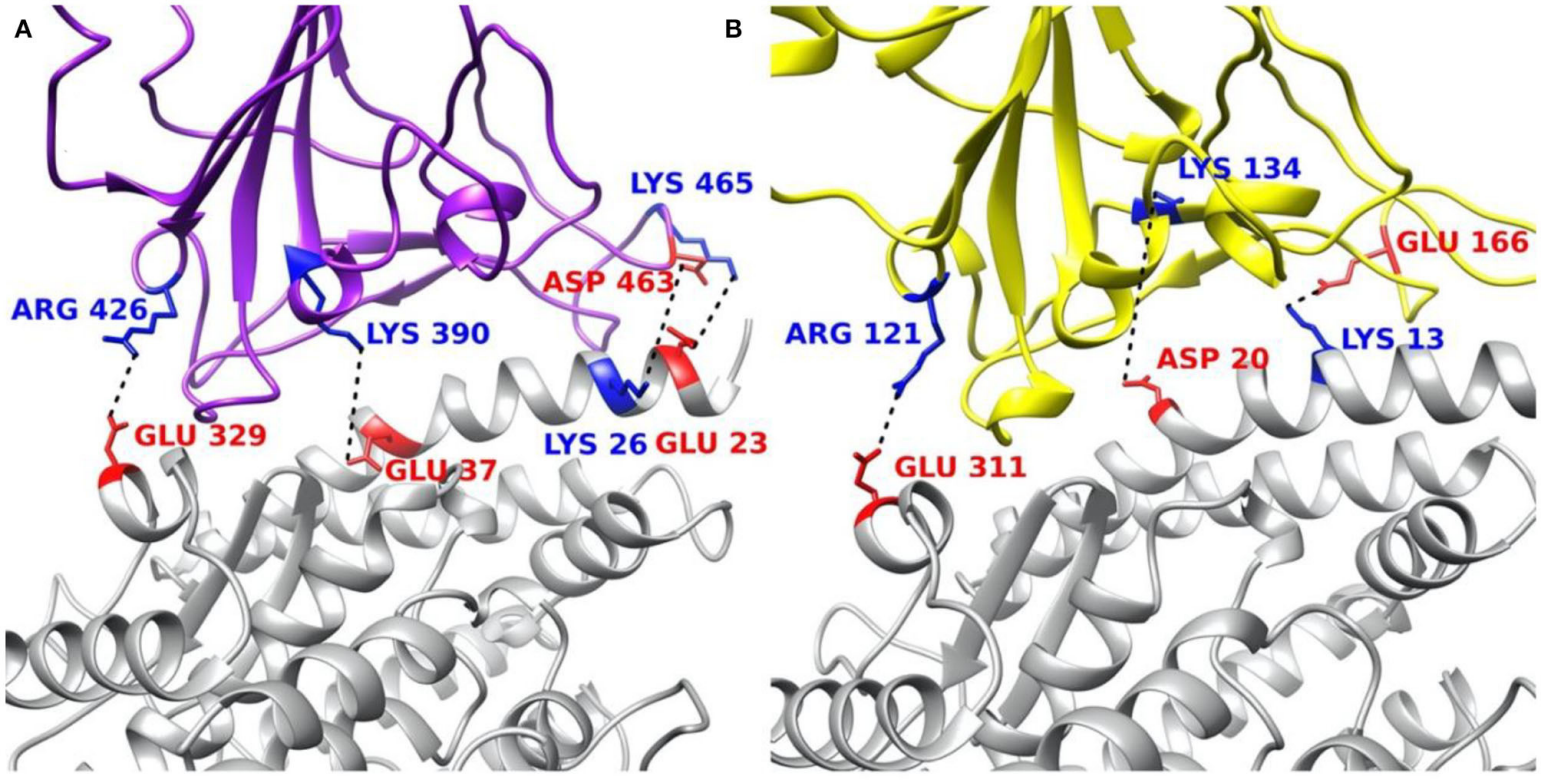
Structural demonstration of key residues that form salt bridges in the interface on both virus S protein RBDs and ACE2 RBD. **(A)** SARS-CoV S protein RBD (purple) and human ACE2 RBD (gray). **(B)** SARS-CoV-2 S protein RBD (yellow) and human ACE2 RBD (gray). Blue stands for positively charged key residues while red represents the negatively charged key residues.

The residues involved in salt bridges and hydrogen bonds are identified as the key residues which may significantly contribute to the binding affinity. Figure 8 illustrates the key residues involved in salt bridges observed. As shown in Figures 8A,B, key residues are mostly around the edges of the binding interfaces rather than the center of the interfaces, and most of the key residues are positive in S protein RBD and negative in ACE2, except for the pair ASP463–LYS26 in SARS-CoV S protein RBD and the special pair GLU166–LYS13 (Supplementary Figure 5) in SARS-CoV-2 S protein RBD. Such salt bridges also play significant roles in binding forces (Figure 5) and electric field line distributions (Figure 4).

## Conclusion

S protein plays a crucial role in SARS-CoV-2 infection by binding to human ACE2. To understand the mechanisms of how S protein binds to ACE2, we compared SARS-CoV with SARS-CoV-2 in biophysical features such as electrostatic binding forces, electric field lines, salt bridges, and hydrogen bonds. We found that even though SARS-CoV and SARS-CoV-2 share very similar structures, there are significant differences in the process when their S proteins bind to ACE2. The common feature is that the calculations of electrostatic surfaces and electric field lines at the binding interfaces demonstrated that ACE2 has a negatively charged binding surface while S protein RBDs are overall positively charged, which provides dominantly attractive forces between ACE2 and S proteins. The differences of electrostatic features between SARS-CoV and SARS-CoV-2 are analyzed in various perspectives as well in this work. Comprehensive analyses were also performed after 100 ns MD simulations, which indicates that SARS-CoV-2 has more high-occupancy (>90%) hydrogen bonds at the interface area between its S protein RBD and ACE2 than SARS-CoV. The electric field line related residues are distributed quite differently, which results in a more robust binding strategy of SARS-CoV-2. Also, the SARS-CoV-2 has higher electric field line density than that of SARS-CoV, which indicates stronger interaction between SARS-CoV-2 and ACE2, compared to that of SARS-CoV. Those facts make the interactions of SARS-CoV-2 more robust than SARS-CoV, which may explain why COVID-19 spreads faster than SARS in 2003. However, this study did not take into account other parts of S proteins except for the binding domain. In this case, we have a hypothesis that the SARS-CoV-2 S protein RBD may be easier to flip out its RBD when reaching out ACE2, since some mutations are found at the linkage area between its binding domain and other parts, and this mechanism may also make the S protein of SARS-CoV-2 easier to bind to ACE2. For this hypothesis, we will study it in our following related work using DelPhiForce Steered Molecular Dynamic (DFMD) approach (Zhang, H. et al., 2020). Based on our 100 ns MD simulations, a list of key residues involved in salt bridges and hydrogen bonds are identified and that may be a supportive reference for future drug development against COVID-19 and other therapeutic research for SARS-CoV-2.

The number of confirmed cases of COVID-19 is still increasing dramatically (Tai et al., 2020). It is highly demanded to reveal the mechanisms of how SARS-CoV-2 infect our human body. This work introduces fundamental binding mechanisms of S proteins binding to ACE2 by using SARS-CoV and SARS-CoV-2 structures. Our approaches involved can be widely utilized to study more viruses in the future, and our findings in this work will also pave the way for other related researches regarding drug designs and treatments for COVID-19 as well as for other coronavirus-caused diseases as well in the future.

## Data Availability Statement

The original contributions presented in the study are included in the article/Supplementary Material, further inquiries can be directed to the corresponding author.

## Author Contributions

YX and LL: conceptualization. YX, DD, and LL: methodology. YX, CK, DD, and HL: validation. YX, JW, and LL: formal analysis. YX and LL: writing-original draft preparation. YX, LL, JW, AS, ST, HL, and QT: writing-review and editing. LL: supervision. All authors have read and agreed to the published version of the manuscript.

## Conflict of Interest

The authors declare that the research was conducted in the absence of any commercial or financial relationships that could be construed as a potential conflict of interest.
