# Supplementary material for: Spike Proteins of SARS-CoV and SARS-CoV-2 Utilize Different Mechanisms to Bind With Human ACE2
Source: Front Mol Biosci. 2020 Dec 9;7:591873. doi: 10.3389/fmolb.2020.591873 (PMC7755986; doi:10.3389/fmolb.2020.591873)
Supplement: Supplementary file 1 [file Presentation_1.pdf]

Spike proteins of SARS-CoV and SARS-CoV-2 utilize different mechanisms to bind with human ACE2

Yixin Xie[1], Chitra B. Karki[1], Dan Du[1], Haotian Li[2], Jun Wang[2], Adebiyi Sobitan[3], Shaolei Teng[3], Qiyi Tang[3], Lin Li[1,2*]

[1]Computational Science Program, University of Texas at El Paso, El Paso, TX.

[2]Department of Physics, University of Texas at El Paso, El Paso, TX.

[3]Department of Biology, Howard University, Washington, D.C.

*** Correspondence:** Lin Li: lli5@utep.edu

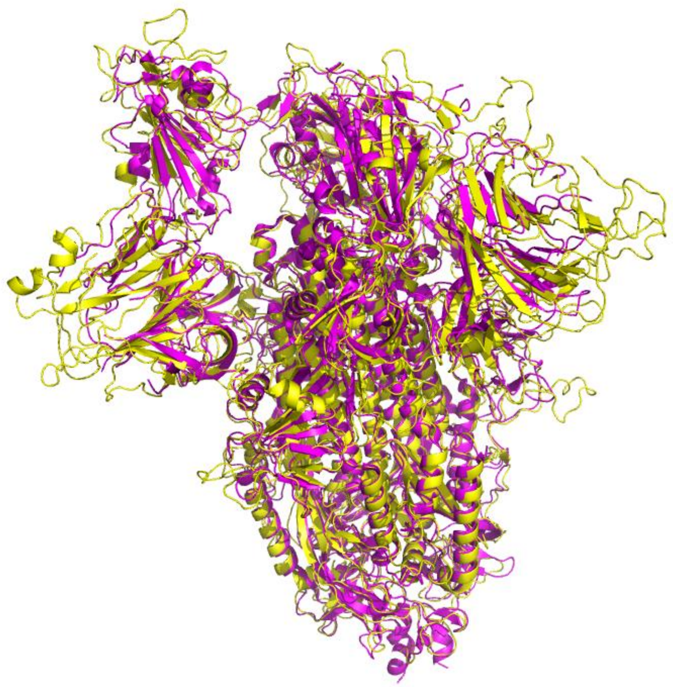

Figure S1. The structural comparison of modeled SARS-COV-2 and its template (6ACG). The template structure is shown in magenta, and the modeled structure is shown in yellow.

```
SARS_CoV        MFIFLLFLTLTSGSDLDRCTTFDDVQAPNYTQHTSSMRGVYYPDEIFRSDTLYLTQDLFL 60
SARS_CoV-2      ------------------CVNLTTRTQLPPAYTNSFTRGVYYPDKVFRSSVLHSTQDLFL 42
                                  *..:        :  .*  *******::***..*: ******

SARS_CoV        PFYSNVTGFHTIN-------HTFGNPVIPFKDGIYFAATEKSNVVRGWVFGSTMNNKSQS 113
SARS_CoV-2      PFFSNVTWFHAIHVSGTNGTKRFDNPVLPFNDGVYFASTEKSNIIRGWIFGTTLDSKTQS 102
                **:**** **:*:       : *.***:**:**:***:*****::***:**:*::.*:**

SARS_CoV        VIIINNSTNVVIRACNFELCDNPFFAVSKPMGT----QTHTMIFDNAFNCTFEYISDAFS 169
SARS_CoV-2      LLIVNNATNVVIKVCEFQFCNDPFLGVYYHKNNKSWMESEFRVYSSANNCTFEYVSQPFL 162
                ::*:**:*****:.*:*::*::**:.*    ..    ::.  ::..* ******:*: *

SARS_CoV        LDVSEKSGNFKHLREFVFKNKDGFLYVYKGYQPIDVVRDLPSGFNTLKPIFKLPLGINIT 229
SARS_CoV-2      MDLEGKQGNFKNLREFVFKNIDGYFKIYSKHTPINLVRDLPQGFSALEPLVDLPIGINIT 222
                :*:. *.****:******** **:: :*. : **::*****.**.:*:*:..**:*****

SARS_CoV        NFRAILTAFS------PAQDIWGTSAAAYFVGYLKPTTFMLKYDENGTITDAVDCSQNPL 283
SARS_CoV-2      RFQTLLALHRSYLTPGDSSSGWTAGAAAYYVGYLQPRTFLLKYNENGTITDAVDCALDPL 282
                .*:::*: .        :.. * :.****:****:* **:***:***********: :**

SARS_CoV        AELKCSVKSFEIDKGIYQTSNFRVVPSGDVVRFPNITNLCPFGEVFNATKFPSVYAWERK 343
SARS_CoV-2      SETKCTLKSFTVEKGIYQTSNFRVQPTESIVRFPNITNLCPFGEVFNATRFASVYAWNRK 342
                :* **::*** ::*********** *: .:*******************:* *****:**

SARS_CoV        KISNCVADYSVLYNSTFFSTFKCYGVSATKLNDLCFSNVYADSFVVKGDDVRQIAPGQTG 403
SARS_CoV-2      RISNCVADYSVLYNSASFSTFKCYGVSPTKLNDLCFTNVYADSFVIRGDEVRQIAPGQTG 402
                :**************: ********** ********:********::**:**********

SARS_CoV        VIADYNYKLPDDFMGCVLAWNTRNIDATSTGNYNYKYRYLRHGKLRPFERDISNVPFSPD 463
SARS_CoV-2      KIADYNYKLPDDFTGCVIAWNSNNLDSKVGGNYNYLYRLFRKSNLKPFERDISTEIYQAG 462
                 ************ ***:***:.*:*:.  ***** ** :*:.:*:*******.  :. .

SARS_CoV        GKPCTP-PALNCYWPLNDYGFYTTTGIGYQPYRVVVLSFELLNAPATVCGPKLSTDLIKN 522
SARS_CoV-2      STPCNGVEGFNCYFPLQSYGFQPTNGVGYQPYRVVVLSFELLHAPATVCGPKKSTNLVKN 522
                ..**.   .:***:**:.***  *.*:***************:********* **:*:**

SARS_CoV        QCVNFNFNGLTGTGVLTPSSKRFQPFQQFGRDVSDFTDSVRDPKTSEILDISPCSFGGVS 582
SARS_CoV-2      KCVNFNFNGLTGTGVLTESNKKFLPFQQFGRDIADTTDAVRDPQTLEILDITPCSFGGVS 582
                :**************** *.*:* ********::* **:****:* *****:********

SARS_CoV        VITPGTNASSEVAVLYQDVNCTDVSTAIHADQLTPAWRIYSTGNNVFQTQAGCLIGAEHV 642
SARS_CoV-2      VITPGTNTSNQVAVLYQDVNCTEVPVAIHADQLTPTWRVYSTGSNVFQTRAGCLIGAEHV 642
                *******:*.:***********:* .*********:**:****.*****:**********

SARS_CoV        DTSYECDIPIGAGICASYHTVSL----LRSTSQKSIVAYTMSLGADSSIAYSNNTIAIPT 698
SARS_CoV-2      NNSYECDIPIGAGICASYQTQTNSPRRARSVASQSIIAYTMSLGAENSVAYSNNSIAIPT 702
                :.****************:* :      **.:.:**:********:.*:*****:*****

SARS_CoV        NFSISITTEVMPVSMAKTSVDCNMYICGDSTECANLLLQYGSFCTQLNRALSGIAAEQDR 758
SARS_CoV-2      NFTISVTTEILPVSMTKTSVDCTMYICGDSTECSNLLLQYGSFCTQLNRALTGIAVEQDK 762
                **:**:***::****:******.**********:*****************:***.***:

SARS_CoV        NTREVFAQVKQMYKTPTLKYFGGFNFSQILPDPLKPTKRSFIEDLLFNKVTLADAGFMKQ 818
SARS_CoV-2      NTQEVFAQVKQIYKTPPIKDFGGFNFSQILPDPSKPSKRSFIEDLLFNKVTLADAGFIKQ 822
                **:********:**** :* ************* **:********************:**

SARS_CoV        YGECLGDINARDLICAQKFNGLTVLPPLLTDDMIAAYTAALVSGTATAGWTFGAGAALQI 878
SARS_CoV-2      YGDCLGDIAARDLICAQKFNGLTVLPPLLTDEMIAQYTSALLAGTITSGWTFGAGAALQI 882
                **:***** **********************:*** **:**::** *:************

SARS_CoV        PFAMQMAYRFNGIGVTQNVLYENQKQIANQFNKAISQIQESLTTTSTALGKLQDVVNQNA 938
SARS_CoV-2      PFAMQMAYRFNGIGVTQNVLYENQKLIANQFNSAIGKIQDSLSSTASALGKLQDVVNQNA 942
                ************************* ******.**.:**:**::*::*************

SARS_CoV        QALNTLVKQLSSNFGAISSVLNDILSRLDKVEAEVQIDRLITGRLQSLQTYVTQQLIRAA 998
SARS_CoV-2      QALNTLVKQLSSNFGAISSVLNDILSRLDKVEAEVQIDRLITGRLQSLQTYVTQQLIRAA
      1002
                ************************************************************

SARS_CoV        EIRASANLAATKMSECVLGQSKRVDFCGKGYHLMSFPQAAPHGVVFLHVTYVPSQERNFT 1058
SARS_CoV-2      EIRASANLAATKMSECVLGQSKRVDFCGKGYHLMSFPQSAPHGVVFLHVTYVPAQEKNFT 1062
                **************************************:*************:**:***

SARS_CoV        TAPAICHEGKAYFPREGVFVFNGTSWFITQRNFFSPQIITTDNTFVSGNCDVVIGIINNT 1118
SARS_CoV-2      TAPAICHDGKAHFPREGVFVSNGTHWFVTQRNFYEPQIITTDNTFVSGNCDVVIGIVNNT 1122
                *******:***:******** *** **:*****:.*********************:***

SARS_CoV        VYDPLQPELDSFKEELDKYFKNHTSPDVDLGDISGINASVVNIQKEIDRLNEVAKNLNES 1178
SARS_CoV-2      V----------------------------------------------------------- 1123
                *

SARS_CoV        LIDLQELGKYEQYIKWPWSHPQFEK 1203
SARS_CoV-2      ------------------------- 1123
```

Figure S2. Genome sequence alignment of SARS-CoV and SARS-CoV-2 S protein RBDs

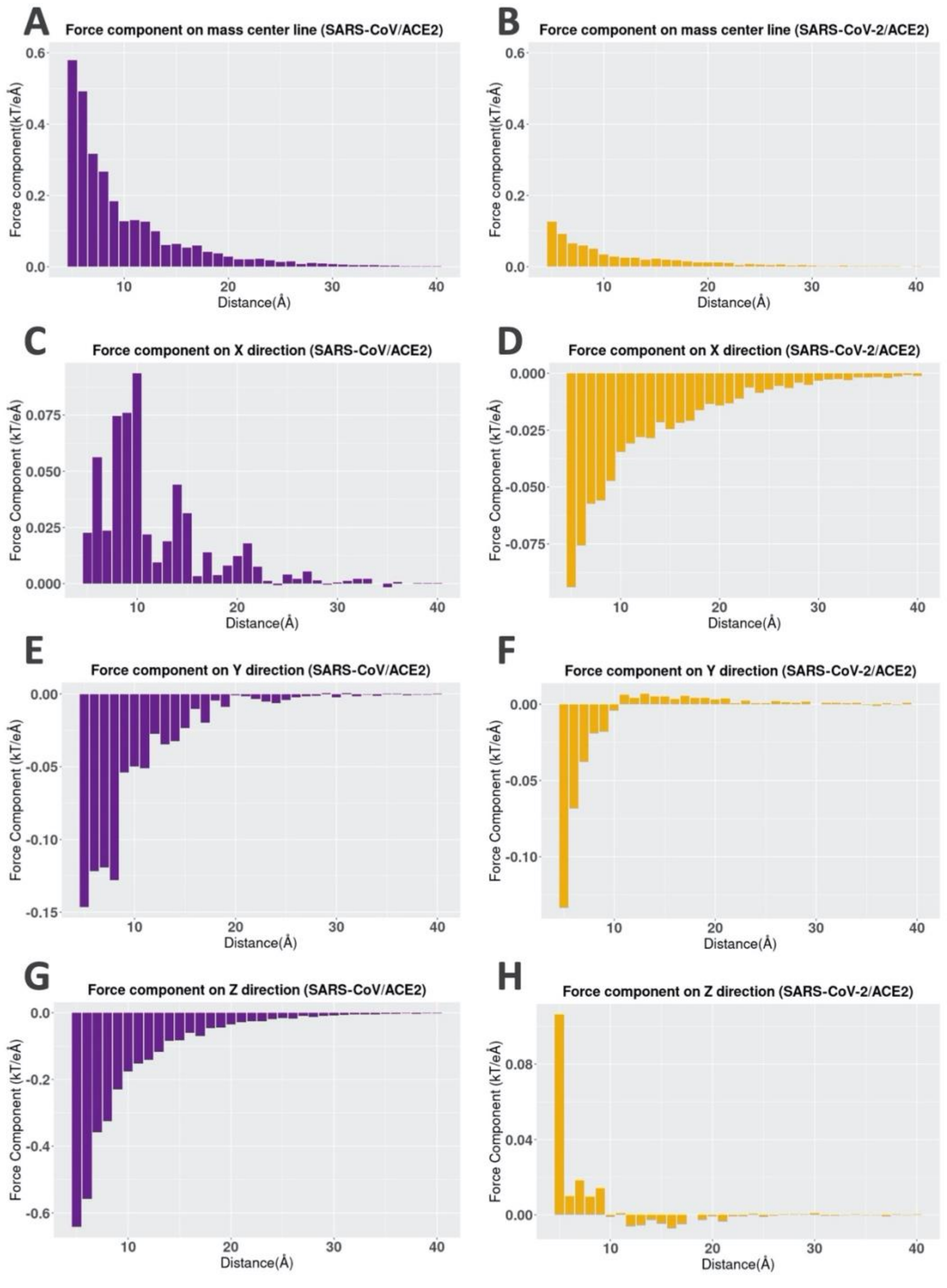

Figure S3. The binding force components on different directions.

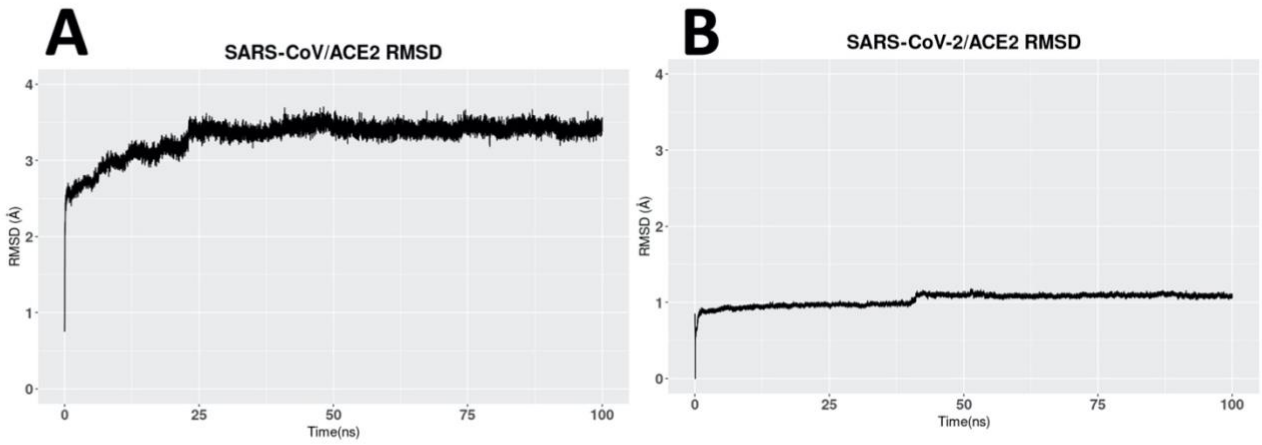

Figure S4. RMSD comparison of SARS-CoV/ACE2 and SARS-CoV-2/ACE2 complex structure

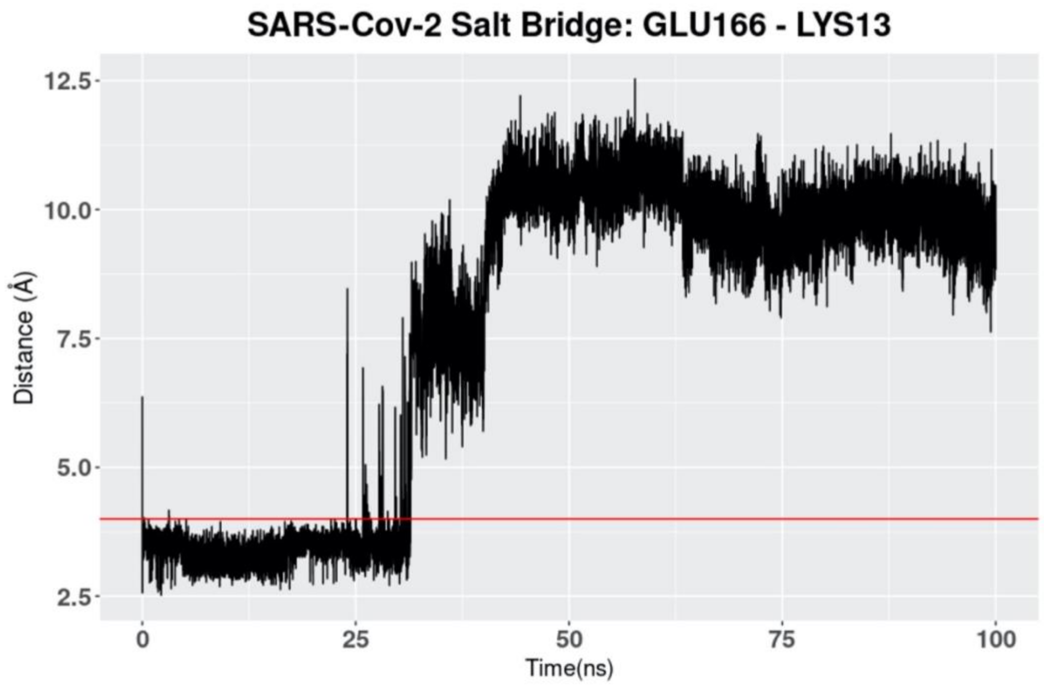

Figure S5. A special salt bridge in SARS-CoV-2 RBD

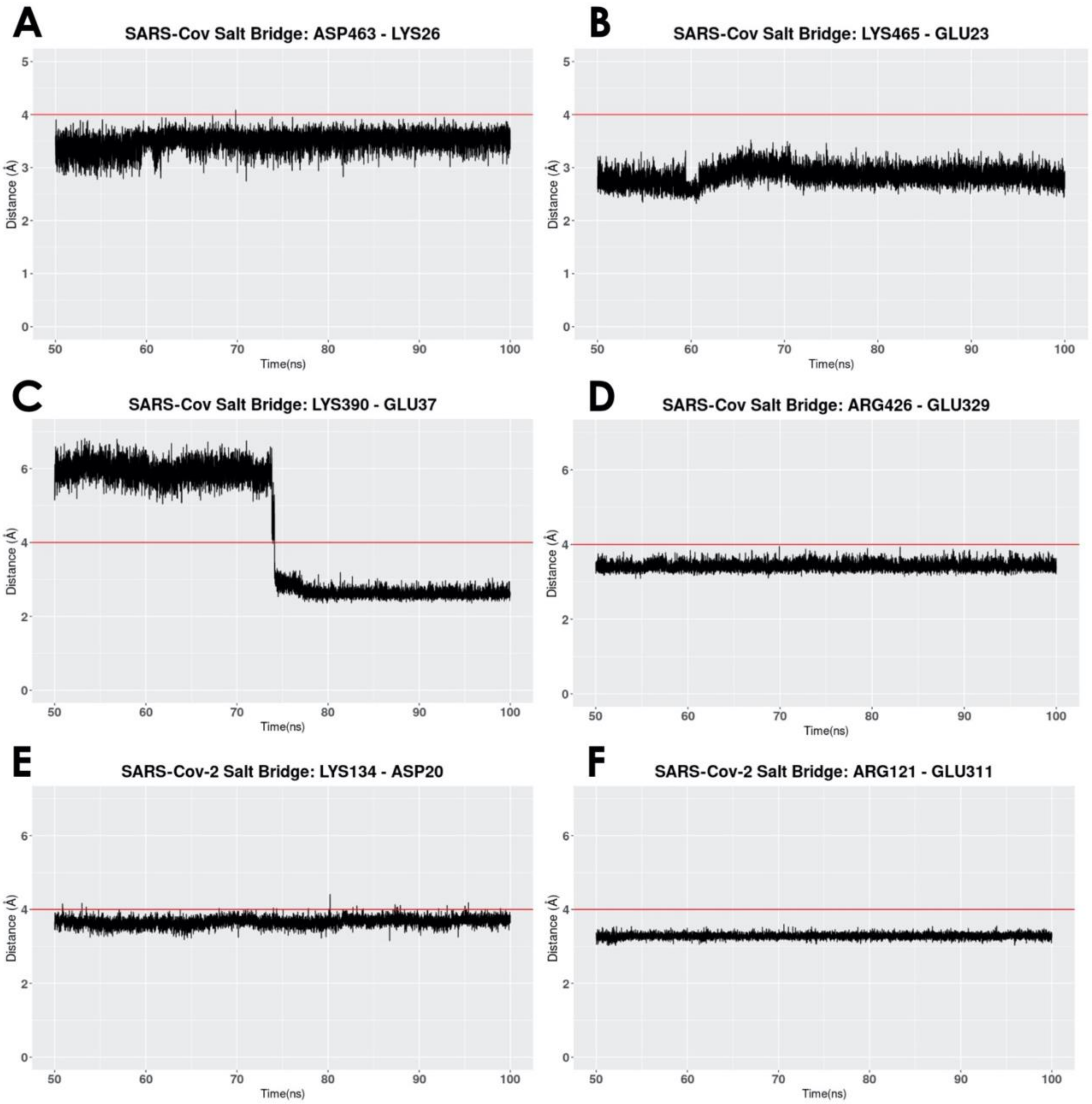

Figure S6. Key residues forming salt bridges at interfaces of S protein RBDs and ACE2 protein binding domain. Red line is the cutoff value of 4 Å which is chosen when calculating the salt bridge. (A) Distance of the ASP463 – LYS26 salt bridge found on the interface of SARS-CoV and ACE2; (B) Distance of the GLU23 – LYS465 salt bridge found on the interface of SARS-CoV and ACE2, (C) Distance of the GLU37 – LYS390 salt bridges found on the interface of SARS-CoV and ACE2; (D) Distance of the GLU329 – ARG426 salt bridges found on the interface of SARS-CoV and ACE2; (E) Distance of the ASP20 - LYS134 salt bridge found on the interface of SARS-CoV-2 and

ACE2; (F) Distance of the GLU311 – ARG121 salt bridges found on the interface of SARS-CoV-2 and ACE2.

Movies:

Movie1: SARS-CoV simulation (100ns)

Movie2: SARS-CoV-2 simulation (100ns)

Movie3: ACE2 electrostatic surface

Movie4: SARS electrostatic surface

Movie5: SARS2 electrostatic surface
